# Acute myocardial infarction following radiofrequency catheter ablation in a child: a case report on the mechanism of coronary artery occlusion assessed by cardiovascular imaging

**DOI:** 10.1093/ehjcr/ytae179

**Published:** 2024-04-17

**Authors:** Ryota Nishio, Shinichiro Doi, Hideo Fukunaga, Tomotaka Dohi

**Affiliations:** Department of Cardiovascular Biology and Medicine, Juntendo University Graduate School of Medicine, 2-1-1 Hongo, Bunkyo-ku, Tokyo 113-0033, Tokyo, Japan; Department of Cardiovascular Biology and Medicine, Juntendo University Graduate School of Medicine, 2-1-1 Hongo, Bunkyo-ku, Tokyo 113-0033, Tokyo, Japan; Department of Pediatrics, Juntendo University Faculty of Medicine, Tokyo, Japan; Department of Cardiovascular Biology and Medicine, Juntendo University Graduate School of Medicine, 2-1-1 Hongo, Bunkyo-ku, Tokyo 113-0033, Tokyo, Japan

**Keywords:** Percutaneous coronary intervention, radiofrequency ablation, intravascular ultrasound, case report

## Abstract

**Background:**

Radiofrequency ablation is a common treatment for atrioventricular nodal re-entrant tachycardia, even in paediatric patients weighing ≥15 kg, where outcomes are similar to those in adults. However, reports of acute coronary artery occlusion after radiofrequency ablation for atrioventricular nodal re-entrant tachycardia are rare.

**Case summary:**

An 11-year-old girl with symptomatic atrioventricular nodal re-entrant tachycardia refractory to drug treatment underwent radiofrequency ablation. During the procedure, ST elevation was observed, and coronary angiography revealed occlusion of the right coronary artery at the segment 4 atrioventricular branch. Intravascular ultrasonography showed a narrowed lumen and an abnormal area of low echogenicity in the adjacent myocardium. After dilation with a 1.5 mm diameter balloon, blood flow was successfully restored. Follow-up coronary computed tomography angiography revealed residual stenosis in the right coronary artery at the segment 4 atrioventricular branch; however, blood flow to the distal occlusion was preserved. The patient was discharged without further complications.

**Discussion:**

To the best of our knowledge, this is the first report of coronary artery occlusion following radiofrequency ablation for atrioventricular nodal re-entrant tachycardia, evaluated using intravascular ultrasonography and coronary computed tomography angiography. Based on the imaging findings, direct thermal injury was considered the cause of occlusion.

**Learning points**

- Based on IVUS findings, we diagnosed the mechanism of coronary artery occlusion due to ablation as direct thermal injury and the oedema.
- This case highlights the importance of prompt intervention for acute coronary artery occlusion associated with RFA for AVNRT in paediatric patients.
- Our report emphasises the need for collaboration between paediatricians and cardiologists to ensure the successful management of such cases.

## Introduction

Radiofrequency ablation (RFA) is an electrophysiological procedure widely used for treating diverse forms of atrial and ventricular arrhythmias. The ablation of atrioventricular nodal re-entrant tachycardia (AVNRT) is an effective antiarrhythmic therapy with a high success rate.^1–3^ In the paediatric population, ablation therapy is increasingly being performed in children weighing ≥15 kg owing to the low risk of complications associated with the procedure.^4,5^ The most common complication of AVNRT ablation is the development of atrioventricular block; however, coronary artery occlusion has rarely been reported.^6–8^ Here, to the best of our knowledge, we describe, for the first time, the use of multimodal cardiovascular imaging to evaluate the mechanism of coronary occlusion after RFA in children.

## Summary figure

**Table:** 

Time period	Event
−1 year	A patient visited our hospital and was diagnosed with supraventricular tachycardia on the electrocardiogram (ECG). Symptomatic supraventricular tachycardia was still observed after the initiation of drug treatment.
−1 month	Our team decided to undergo radiofrequency ablation (RFA).
Day 0	The patient was admitted to our hospital.
Day 1	Electrophysiological studies detected slow–fast atrioventricular nodal re-entrant tachycardia (AVNRT). Our team performed ablation in the posterior septum. After ablation, ST elevation was detected on leads II, III, and aVF on ECG. Coronary angiography revealed occlusion of the right coronary artery at the segment 4 atrioventricular branch (4AV). Good blood flow was achieved by dilation with a balloon, and there was also an improvement in ST elevation on ECG.
Day 3	Our team administered nicorandil and heparin intravenously for two days Troponin T and creatine kinase MB peaked out.
Day 7	Coronary computed tomography angiography (CCTA) revealed blood flow to the distal occlusion was preserved.
Day 10	Discharged from our hospital with aspirin, nicorandil, and colchicine.
Day 30	Continued outpatient follow-up without any episodes of arrhythmia or angina.
Day 90	The cardiopulmonary exercise test did not reveal any findings of arrhythmia or myocardial ischemia.

## Case presentation

An 11-year-old female patient weighing 32.1 kg presented with palpitations and symptomatic supraventricular tachycardia resistant to verapamil, flecainide, and bisoprolol fumarate. Physical examination demonstrated no signs of cardiac congestion, such as jugular vein distension or lower leg oedema. Heart auscultation revealed no murmurs. The electrocardiogram (ECG) showed sinus rhythm without significant ST-segment changes, and the echocardiogram revealed a left ventricular ejection fraction of 70%, and no anatomical abnormalities were identified. Our team decided to perform RFA under intravenous anaesthesia after informed consent was obtained from the patient’s parents. Heparin sodium was administered and activated clotting times were maintained above 250 s throughout the procedure. Electrophysiological studies have shown evidence of slow–fast AVNRT. Three attempts at slow-pathway area ablation were made using an ablation catheter (THERMOCOOL SMARTTOUCH DF curve, Biosense Webster, Inc., CA, USA) placed in the posterior septum. Ablation was performed at an initial power of 25 W for 40 s, followed by two additional applications at 30 W for 30 s each.

**Figure 1:**
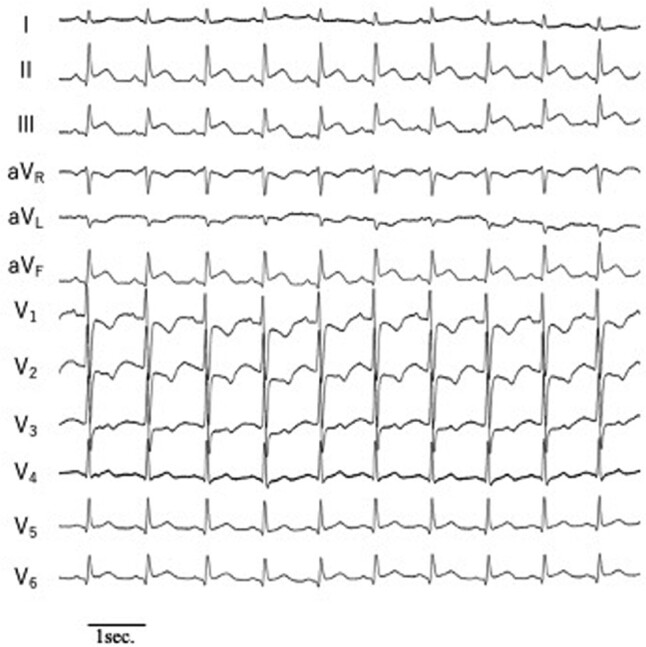
Electrocardiogram showing ST elevation on leads II, III, and aVF.

**Figure 2:**
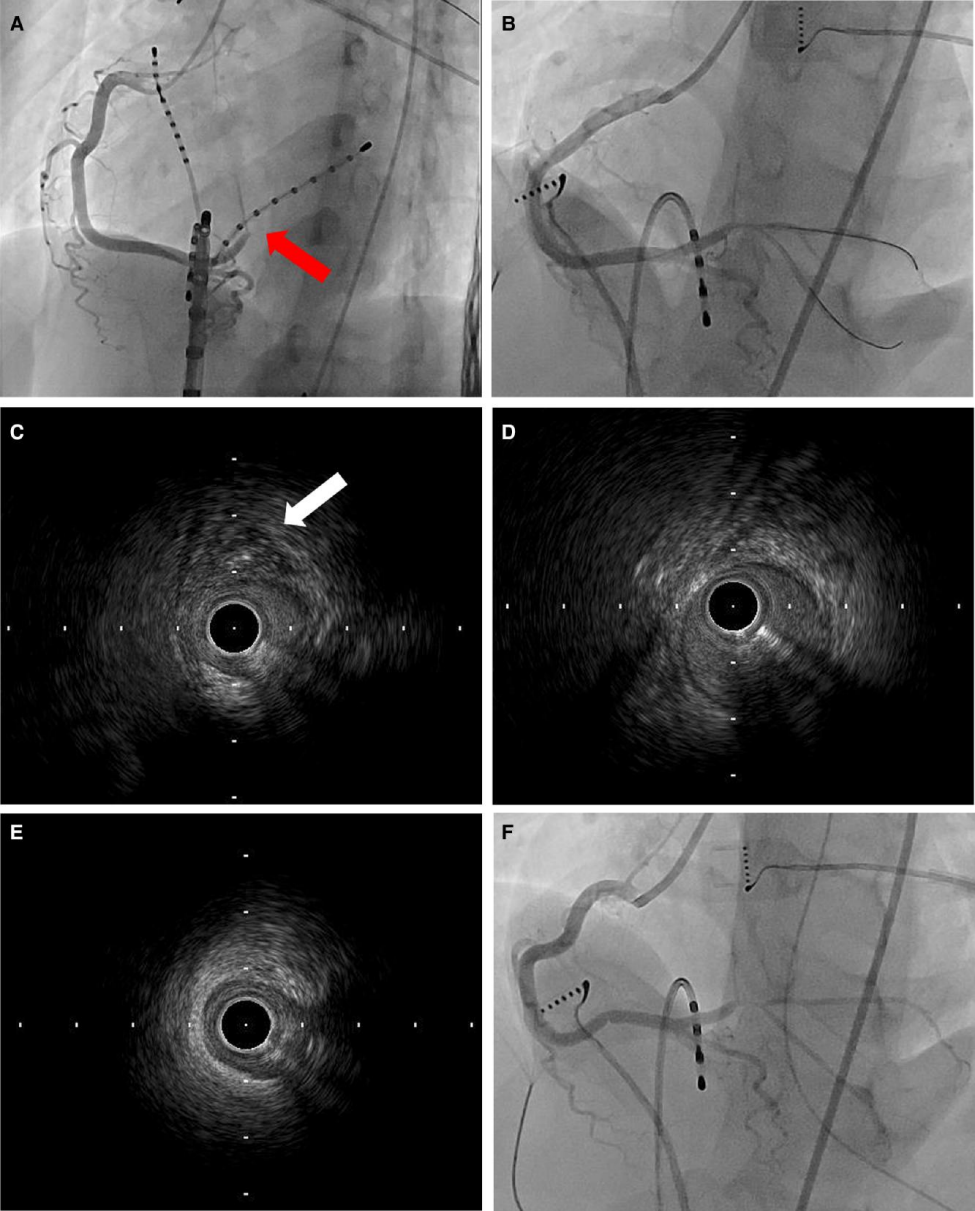
Coronary angiography and intravascular ultrasound imaging. (*A*) Occlusion of the dominant right coronary artery at the segment 4 atrioventricular branch (arrow). (*B*) Revascularisation following the passage of a floppy wire. (*C*) The vessel is narrowed at the target lesion. An abnormal area of low echogenicity is detected in the adjacent myocardium (arrow). (*D*) The proximal site shows no evidence of plaque rupture and maintains a normal three-layered structure. (*E*) Distal site maintained a normal structure. (*F*) Final angiography.

After ablation, ST elevation was detected on leads II, III, and aVF on electrocardiography (*Figure 1*). Immediate coronary angiography revealed occlusion of the dominant right coronary artery at the segment 4 atrioventricular branch (4AV) (*Figure 2A*). Intracoronary isosorbide dinitrate (0.5 mg/time) and nicorandil (1.2 mg/time) were administered multiple times with no effect. A floppy wire was passed through the lesion, and thrombus aspiration was performed; however, the thrombus was not aspirated. Reperfusion was achieved using a floppy wire as a guide (*Figure 2B*), and intravascular ultrasound (IVUS) was performed. Intravascular ultrasound findings revealed a preserved normal structure in the occluded segment of the coronary artery, although the vessel had a narrowed lumen. An abnormal area of low echogenicity was detected in the adjacent myocardium (*Figure 2C*). Intravascular ultrasound findings showed no evidence of plaque rupture, thrombus, haematoma, coronary artery dissection, or coronary artery spasm at the occlusion site, as well as proximal and distal to the occlusion site; however, they revealed haziness due to slow blood flow caused by stenosis. (*Figure 2D* and *E*). Despite our attempt to discontinue the procedure owing to the vessel narrowness and successful revascularisation, re-occlusion was detected upon the removal of the floppy wire. However, upon floppy wire removal, good blood flow was achieved by dilation with a 1.5 mm diameter balloon (*Figure 2F*), and ST elevation on electrocardiography also improved.

**Figure 3:**
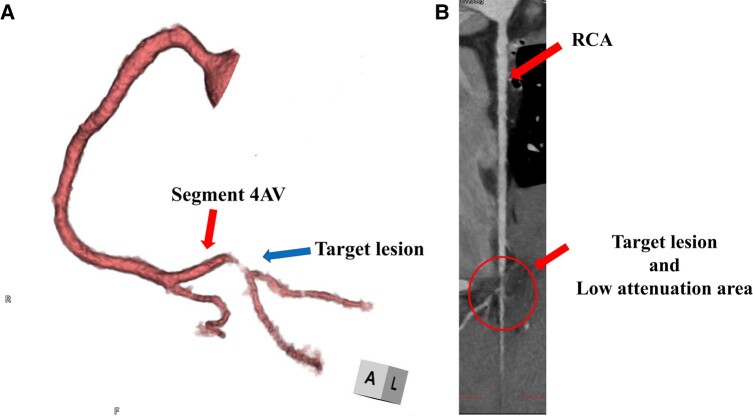
(*A*) Coronary computed tomography angiography indicating residual stenosis (arrow) in the right segment 4 atrioventricular branch (arrow); however, blood flow to the distal occlusion is preserved. (*B*) On coronary computed tomography, the myocardial region surrounding the culprit lesion demonstrates an area of low attenuation compared to that of the normal myocardium.

**Figure 4:**
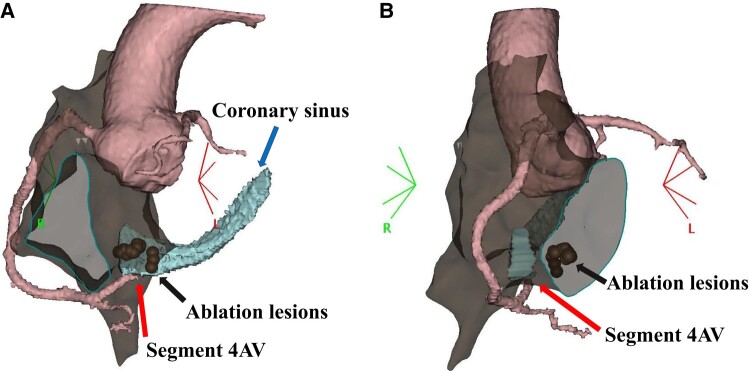
The combined electro-anatomical and coronary computed tomography angiography image (*A* and *B*). Ablation lesions and stenosis of the segment 4 atrioventricular branch are located near each other. (*A)* Left anterior oblique view. Ablation lesions (dots indicated by arrows), stenosis of segment 4 atrioventricular branch (arrow), and the coronary sinus (arrow). (*B*) Right anterior oblique view. Ablation lesions (dots indicated by arrows) and stenosis of segment 4 atrioventricular branch (arrow).

The patient was transferred to the coronary care unit and administered a nicorandil drip at 48 mg/day and a heparin drip at 10 000 units/day for 2 days. Maximum values of 1.31 ng/mL serum troponin T (reference range: <0.1 mg/mL) and 56 U/L creatine kinase MB (reference range: <12 U/L) were observed. No further ST-segment changes or Q waves were found on the resting ECG. Coronary computed tomography angiography (CCTA) was performed 1 week later. CCTA revealed residual stenosis in the right coronary artery at the segment 4AV; however, blood flow to the distal occlusion was preserved (*Figure 3A*). The myocardial region surrounding the culprit lesion exhibited an area of low attenuation compared with that of the normal myocardium (*Figure 3B*). In the image that combined the electro-anatomical view with CCTA, the ablation lesions of the posterior septum and occlusion site of segment 4AV were found to be in close proximity (*Figure 4A* and *B*). Ten days later, the patient was discharged from the hospital on aspirin 100 mg p. o. QD, nicorandil 5 mg p. o. BID, and colchicine 0.25 mg p.o. QD. No arrhythmia episodes were observed after discharge, and the cardiopulmonary exercise test performed 3 months post-discharge revealed no findings of arrhythmia or myocardial ischaemia. Considering the risk of restenosis, coronary angiography was performed 5 months later. Since coronary flow was preserved and no symptoms of angina were observed, a follow-up observation approach was adopted.

## Discussion

Coronary artery injury during RFA is rare (0.1%) but can have serious consequences, particularly during posteroseptal accessory pathway ablations and cavotricuspid isthmus ablation, where right coronary artery and left circumflex branch injuries frequently occur.^9^ The mechanism of coronary artery occlusion after RFA involves extrinsic compression from surrounding oedema, coronary vasospasm, air embolisation, plaque rupture, arterial dissection, and direct thermal injury to the artery, resulting in ablation energy.^7,10^ In this case, IVUS findings showed no evidence of plaque rupture, thrombus, haematoma, coronary artery dissection, or thickening of the intima-media, which is characteristic of coronary spasm.^11^ The target vessel had a narrow lumen, and an abnormal hypoechoic area was detected in the adjacent myocardium. The location of the hypoechoic area was consistent with the septal region based on its position in relation to the epicardium. Thus, the hypoechoic area could be attributed to oedema caused by direct thermal injury from the posterior septum. It was hypothesised that the mechanism of vascular occlusion was the compression of the coronary artery by perivascular oedema. Our team decided not to proceed with stent placement due to the patient’s young age, the risks associated with dual antiplatelet therapy, and the expectation of improvement in perivascular oedema.

One of the risk factors for coronary artery injury due to ablation is the distance between the coronary artery and the ablation site. If the distance between the ablation site and the coronary artery is <2 mm, there is a high risk of coronary artery injury.^10,12,13^ In this case, the distance between the coronary artery and the posterior septum was 2.4 mm, as measured using CCTA. Possible causes of direct thermal injury in this case, besides the distance between the coronary artery and the ablation site, may include the patient’s age, as paediatric patients have lesser pericardial fat than adults, as well as a narrower diameter of the coronary artery and slower blood flow, which may have hindered the dissipation of thermal energy into the surrounding coronary vessels. As preventive measures, reducing the power output, contact force and duration of energy application during ablation, as well as lowering the ablation index may be considered. However, few reports exist on coronary artery occlusion due to ablation in paediatric patients to confirm these hypotheses; thus, further investigation is required.

This case highlights the importance of prompt intervention for acute coronary artery occlusion associated with RFA for AVNRT in paediatric patients. Based on the IVUS findings, the direct thermal injury was considered the cause of the occlusion. Our report emphasises the need for collaboration between paediatricians and cardiologists to ensure the successful management of such cases.

## Data Availability

The data that support the findings of this study are available from the authors upon reasonable request.

## Lead author biography

Ryota Nishio works as Cardiologist at the Department of Cardiovascular Biology and Medicine, Juntendo University Graduate School of Medicine in Japan. His research interest is mainly focused on coronary artery disease and structure heart disease. He loves his daughters ‘midori’ and ‘aoi’.
